# Patients Presenting with Bull-related Injuries to a Southern Indian Emergency Department

**DOI:** 10.5811/westjem.2020.5.47212

**Published:** 2020-09-25

**Authors:** Subhathra Nagarajan, Narendra Nath Jena, Kevin Davey, Katherine Douglas, Jeffrey Smith, Janice Blanchard

**Affiliations:** * Meenakashi Mission Hospital, Department of Emergency Medicine, Madurai, Tamil Nadu, India; † George Washington University, Department of Emergency Medicine, Washington, District of Columbia

## Abstract

**Introduction:**

Bull-related injuries are commonly observed in rural areas of India as result of the animal’s use in sporting events as well as for agricultural purposes. These patients need early resuscitation due to complications from severe injuries. Previous work examining the epidemiology of bull-related injuries is limited, with most studies focusing on injuries in Spain and Latin America. There is scant literature examining the prevalence of such injuries in India. The objective of this study was to evaluate the demographic and clinical characteristics of bull-related injuries at a hospital in Tamil Nadu, India.

**Methods:**

This was a prospective, observational study of patients who presented to an emergency department (ED) in Madurai, India, with a reported history of bull-related injuries between June 2017 and March 2019. We recorded information about patient demographics, location of injury, disposition, initial Injury Severity Score (ISS), and transport time.

**Results:**

Our sample included a total of 42 patients. Almost a third of patients who presented were between the ages of 20–30 years (31%, n = 13), and most were male (86%, n = 36). Approximately 59% of patients (n = 25) had provoked injuries, occurring as a result of active participation during sporting activities. Injuries to the trunk were most common (55%, n = 23), followed by injuries to the perineum (19%, n = 19). The majority of patients (59.5%) had penetrating injuries (n = 25), The mean ISS was 10.1 (standard deviation 6.3). Five (12%) patients had a complication after injury including intra-abdominal abscess formation, peritonitis, and sepsis. Two patients died as a result of septicemia from peritonitis.

**Conclusion:**

Bull-related injuries may result in significant morbidity and mortality. Education of the population about the dangers of bull injuries from sporting events and the need for early transportation to the ED have the potential for significant reduction in morbidity and mortality.

## BACKGROUND

Bull-related injuries are commonly seen in Tamil Nadu, India, due to the frequent use of bulls in daily agricultural activities as well as in sporting events. Jallikattu, a popular sport in Madurai, Tamil Nadu, is practiced during the Mattu Pongal celebration, which honors the role of cattle in supporting the livelihood of Indian farmers. As part of this event, the bull is released into a confined area and participants alternate attempts to stop its movement by embracing its hump.^1^ Most of these events occur in rural areas, where emergency care is scarce. When care is available there may be prolonged transport times to reach acute care services, leading to adverse medical outcomes.^2^

Injuries sustained from bulls are extensive and often result in prolonged hospitalization. Most bull injuries are penetrating, occurring as a result of direct goring from the horn.^2^,^3^ Blunt injuries can occur as a result of the force sustained from impact with the ground after being thrown from the bull.^2^,^3^ The size and contamination of bull horns complicate penetrating injuries due to a higher incidence of wound infection and delayed healing.^3^

The majority of studies published about the epidemiology of bull-related injuries are from Spain and Latin America, focusing on trauma resulting from bull fights.^3^–^8^ While there have been some reports from India,^3^,^9^,^10^ less is known about the initial presentation to the emergency department (ED). In particular, little is known about complicating factors such as transport time that may lead to delays in care. This is a challenge that makes the presentation of bull injuries unique to low-resource settings such as India, compared to that of areas such as Spain, where there may be better access to emergency care.^1^ Because bull-related injuries involve the need for aggressive and early resuscitation in the ED, it is important to understand the mechanism and consequences of such injuries in order to provide timely management.

## METHODS

We collected data prospectively from all patients who presented with bull-related injuries to a South Indian ED in Madurai, Tamil Nadu, between June 2017–March 2019. The ED is one of the largest in Tamil Nadu and has a residency training program along with surgical specialty services. Using the ED health chart, we recorded information about patient demographics, location of injury, disposition, initial Injury Severity Score (ISS) and transport time. We examined the association between disposition and ISS and transport time for all patients in our sample. We calculated the mean, standard deviation, and p values between groups by disposition using chi-squared analysis for discrete variables and t test for continuous variables with SDSS software, version 20.0. A p value < 0.05 was considered significant. The study was approved by our hospital’s institutional review board.

## RESULTS

During our study time period 42 patients presented to the ED with a bull-related injury. Patient demographics are shown in Figure. Almost a third of patients who presented were between the ages of 20–30 years (31%, n = 13), and most were male (86%, n = 36). Approximately 59% of our patients (n = 25) had provoked injuries, occurring as a result of active participation during Jallikattu sporting activities. The remaining cases were unprovoked, sustained either as spectators during a sporting event or through domestic work. The average time between injury and presentation to the ED (transport time) was 2.88 hours (standard deviation [SD] 2.9 hours).

**Figure 1:**
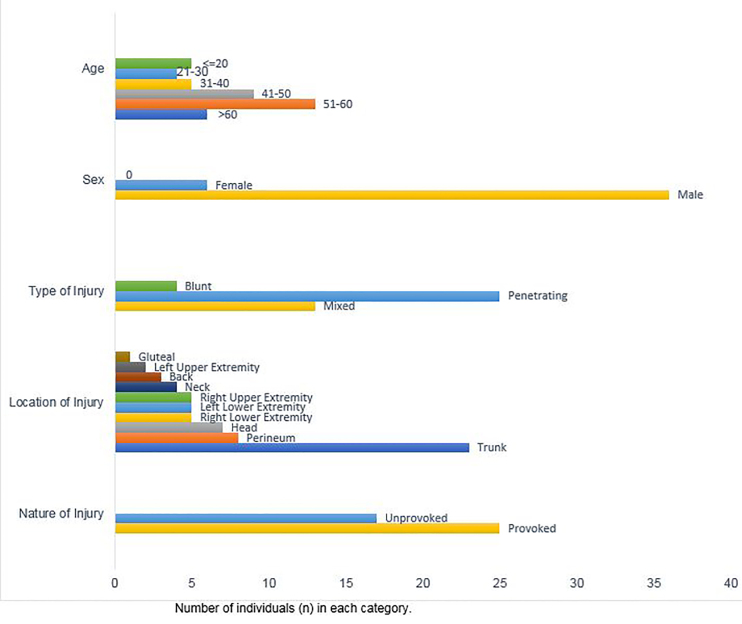
Sample demographics of patients presenting with bull-related injuries in the state of Tamil Nadu, India.

Injuries to the trunk were most common (55%, n = 23), followed by injuries to the perineum (19%, n = 19). The majority of patients (59.5%) had penetrating injuries (n = 25), approximately 31% (n = 13) had blunt injuries, and almost 9.5% (n = 4) had both blunt and penetrating injuries (See Figure 1).

Table 1 shows the injury type and disposition of patients in our sample. Of patients with abdominal injuries, eight had solid organ injuries including one with a grade 2 splenic laceration, three with liver lacerations (grades 1, 3 and 5), and one with a grade 4 renal injury. Three had small bowel perforations. Of patients with trunk injuries, seven required chest tubes for pneumothorax or hemothorax. Eighteen patients had fractures involving the cervical spine (n = 3, 7.1%), ribs (n = 6, 14.2%), extremities (n = 4, 9.6%), and maxillofacial bones (n = 1, 2.4%). The most common procedures performed were laceration repair either under local (n = 7, 16.7%) or general anesthesia (n = 9, 21.4%), laparotomy (n = 7, 16.7%), and chest tube placement (n = 6, 14.2%). The mean transport time to the hospital was 2.88 hours (SD 2.9 hours). Thirty-two patients (76%) were admitted to the intensive care unit (ICU); all other patients were admitted to the ward. The mean ISS was 10.1 (SD 6.3) with a mean hospital length of stay of 6.55 days (SD 4.9 days).

Table 2 shows differences between patients admitted to the ICU and the ward. Patients who had a trunk injury were more likely to require an ICU admission than those who sustained injuries to other locations of the body (65.5% vs 34.4%, p = 0.01). Patients with a higher ISS were more likely to require an ICU admission (mean ISS among ICU admitted patients was 11.75 vs 4.8 in ward patients [p<0.001]). Five (12%) patients had complications during their hospital stay including intra-abdominal abscess formation, peritonitis, and sepsis. Two (5%) patients died during hospitalization as a result of septicemia from peritonitis. The hospital course of all of the remaining patients in our sample was uneventful and no major complications were reported.

Patients who were admitted to the ICU had a longer mean transport time than those admitted to the ward, but this difference was not statistically significant. The mean transport time for patients who were admitted to the ICU was 3.02 hours (SD 3.1 hours) and that of patients admitted to the ward was 2.41 hours (SD 1.7 hours). The two patients who died had a much longer mean transport time (mean 14.2 hours, SD 0.2 hours) and a higher mean ISS (25, SD 0.1) as compared to survivors (mean transport time 2.3 hours, SD 1.4; and mean ISS 9.4, SD 4.4, p<0.001.)

## DISCUSSION

Our sample shows similar patterns of injury as seen in other regions. Studies of injuries involving bulls for agricultural and sporting activities in the United States and India show the predominance of abdominal and perineal injuries.^8^–^11^ In studies of professional bullfighters, injury patterns are somewhat different.^12^ In a review of 68 cases of professional bullfighters in Mexico, upper and lower extremity injuries were most common (66%) followed by injuries of the perineum.^7^ In a series from Spain and southern France, penetrating extremity injuries accounted for 75% of cases in 317 individuals.^5^

Patients who present to the ED with bull-related injuries are at significant risk for high morbidity and mortality. Although most patients in our sample survived their injuries, the two who died had a significantly longer mean transit time between the site of the occurrence of injury and the ED. In India, emergency medicine is still in its nascency and prehospital care services are limited, particularly in rural areas where bull-related injuries are most likely to occur.^8^–^11^ This may have contributed to the long mean transport time observed in our sample.

Although bull-related injuries are a small proportion of all traumas in Southern India it is still a significant source of morbidity. In one series from a Tamil Nadu ED, animal-related injuries accounted for less than 1% of traumas, compared to 65% from motor vehicle accidents (although that study did not specifically evaluate bull-related injuries).^13^ More research is needed on a larger sample of patients to better understand the prevalence of bull-related injuries and to design strategies aimed at prevention and management of such injuries. This includes the training of specialists who treat these injuries as in Spain and Mexico.^7^ In addition, while the Indian government has issued some regulations of the sport of Jallikattu, additional intervention may be needed to insure that safety measures are practiced.^1^

## LIMITATIONS

There were a number of limitations to our study. This was a small sample at a single center in India. Results may not be generalizable to the rest of the population of India, particularly at hospitals that do not have emergency medical care. We did not have information on any interventions received prior to arriving to the ED or comorbid conditions that may have impacted outcomes. In addition, we did not follow patients for delayed adverse events that occurred after hospital discharge. We also did not have information about deaths occurring outside of the hospital setting or about patients who did not present to our ED; therefore, our results may have been skewed in favor of patients with greater or lower severity as compared to those who presented elsewhere or who did not present to any ED.

## CONCLUSION

Despite the occurrence of bull-related injuries in India, there is little awareness about the dangers of using bulls for sport, offering opportunities for public health outreach about the prevention of such injuries and the need for early intervention. Because the sport of Jallikattu is an integral part of culture in Tamil Nadu, there are ample opportunities for community outreach in this population that have the potential for significant reduction in morbidity and mortality.

## Figures and Tables

**Table 1:** Injury, procedures, and disposition (n = 42) in patients treated for bull-related injuries.

	n (%)
Injuries	
Contusion/abrasion	15 (35.7)
Laceration	
Superficial (< =1 cm)	5 (12.0)
Deep (>1 cm)	22 (52.4)
Closed head injury	2 (4.8)
Solid organ/bowel injury	
Liver laceration	3 (7.1)
Splenic laceration	1 (2.4)
Renal injury	1 (2.4)
Bowel perforation	3 (7.1)
Fractures	
Maxillofacial fracture	1 (2.4)
Extremity fractures	4 (9.6)
Spine fractures	3 (7.1)
Rib fracture	6 (14.2)
Clavicle	2 (4.8)
Procedures performed	
Conservative management	8 (19.2)
Laceration/wound closure local anesthesia	7 (16.7)
Kyphoplasty/cord decompression	2 (4.8)
Wound repair under general anesthesia/hematoma evacuation	9 (21.4)
Fracture reduction/fixation	4 (9.5)
Craniotomy	1 (2.4)
Laparotomy/peritoneal drain	7 (16.7)
Perineal wound exploration/repair general anesthesia	3 (7.1)
Chest tube	6 (14.2)
Wound debridement/closure	4 (9.5)
Disposition	
Admission to ICU	32 (76.2)
Admission to ward	10 (23.8)
Hospital length of stay (days)	6.55 (SD 4.9)
Mean Injury Severity Score	10.1 (SD 6.3)
Mean time between injury and ED presentation (transit time in hours)	2.88 (SD 2.9)

*cm*, centimeter; *ICU*, intensive care unit; *ED*, emergency department; *SD*, standard deviation.

**Table 2:** Relationship between characteristics of bull-related injury and disposition.

	Intensive Care Unit N = 32	Ward N = 10	P-value
Location of injury			
Trunk	21 (65.6%)	2 (20.0%)	0.01
Other	11 (34.4%)	8 (80.0%)	0.01
Transport time (hours)	3.02 hours (SD 3.1)	2.41 hours (SD 1.7)	0.56
Injury Severity Score	11.75 (SD 6.3)	4.8 (SD 1.8)	<0.001

*SD*, standard deviation.
